# Pre-clinical characterization of GMP grade CCL21-gene modified dendritic cells for application in a phase I trial in Non-Small Cell Lung Cancer

**DOI:** 10.1186/1479-5876-6-38

**Published:** 2008-07-22

**Authors:** Felicita Baratelli, Hiroko Takedatsu, Saswati Hazra, Katherine Peebles, Jie Luo, Pam S Kurimoto, Gang Zeng, Raj K Batra, Sherven Sharma, Steven M Dubinett, Jay M Lee

**Affiliations:** 1 UCLA Lung Cancer Research Program of the Jonsson Comprehensive Cancer Center, Division of Pulmonary and Critical Care Medicine, Department of Medicine, Los Angeles, CA 90095, USA; 2 Department of Pathology and Laboratory Medicine, Geffen School of Medicine at UCLA, Los Angeles, CA, 90095, USA; 3 Molecular Medicine Laboratory, Veteran's Affairs Greater Los Angeles Healthcare System, Los Angeles, CA 90073, USA; 4 Department of Urology, Geffen School of Medicine at UCLA, Los Angeles, CA 90095, USA; 5 Division of Cardiothoracic Surgery, Department of Surgery, David Geffen School of Medicine at UCLA, Los Angeles, CA 90095, USA

## Abstract

**Background:**

Our previous studies have demonstrated that transduction of human dendritic cells (DC) with adenovirus encoding secondary lymphoid chemokine, CCL21, led to secretion of biologically active CCL21 without altering DC phenotype or viability. In addition, intratumoral injections of CCL21-transduced DC into established murine lung tumors resulted in complete regression and protective anti-tumor immunity. These results have provided the rationale to generate a clinical grade adenoviral vector encoding CCL-21 for *ex vivo *transduction of human DC in order to assess intratumoral administration in late stage human lung cancer.

**Methods:**

In the current study, human monocyte-derived DC were differentiated by exposure to GM-CSF and IL-4 from cryopreserved mononuclear cells obtained from healthy volunteers. Transduction with clinical grade adenoviral vector encoding CCL21 (1167 viral particles per cell) resulted in secretion of CCL21 protein.

**Results:**

CCL21 protein production from transduced DC was detected in supernatants (24–72 hours, 3.5–6.7 ng/4–5 × 10^6 ^cells). DC transduced with the clinical grade adenoviral vector were > 88% viable (n = 16), conserved their phenotype and maintained integral biological activities including dextran uptake, production of immunostimulatory cytokines/chemokines and antigen presentation. Furthermore, supernatant from CCL21-DC induced the chemotaxis of T2 cells *in vitro*.

**Conclusion:**

Viable and biologically active clinical grade CCL21 gene-modified DC can be generated from cryopreserved PBMC.

## Background

Lung cancer is the leading cause of cancer-related death in the United States with a 5-year survival rate of only 15% [1]. Thus, development of new therapeutic strategies is required. The potential for the immune system to induce tumor regression has stimulated much research into development of vaccines to unmask tumor antigens, leading to a specific host immune response against the tumor [2]. However, the poor immunogenicity of human lung cancer due to low expression of major histocompatibility complex (MHC) antigens, a deficit in transporter-associated with antigen-processing, and lack of co-stimulatory molecules, have rendered most of the immunotherapeutic efforts ineffective [3]. In addition, tumor cell-derived inhibitory factors and immune suppressive cells such as T regulatory cells also impede the immune response to Non-Small Cell Lung Cancer (NSCLC) [4-7].

Dendritic cells (DC) are the most potent antigen presenting cells (APC) capable of inducing primary immune responses [8]. DC express high levels of MHC and costimulatory molecules such as CD40, CD80, and CD86. DC also produce high levels of cytokines and chemokines, attracting antigen-specific T cells *in vivo*. These properties, combined with the efficient capture of antigens by immature DC, allow them to efficiently present antigenic peptides and costimulate antigen-specific naïve T cells [8]. Presentation of tumor-associated antigens by DC and their recognition by cytotoxic T lymphocytes (CTL) play an important role in the eradication of tumor cells [9]. Based upon the importance of DC in tumor immunity, a variety of strategies have been used to exploit this cell type in cancer-immunotherapy [10-12]. Advances in the isolation and *in vitro *propagation of DC combined with identification of specific tumor antigens have allowed initiation of clinical trials testing DC-based vaccines [10-12]. DC transfer has been demonstrated to be a safe approach in clinical studies [13-16].

Strategies employing DC in immunotherapy have included pulsing isolated DC with tumor antigen peptides, apoptotic tumor cells, or tumor lysates *ex vivo *[17-19]. DC have also been genetically modified with genes encoding tumor antigens or immunomodulatory proteins [20-22]. There is evidence that DC transduced with adenoviral vectors (AdV) have prolonged survival and resistance to spontaneous and Fas-mediated cell death [23]. This could result in the improved delivery of immunotherapy. AdV transduction itself can also augment the capacity of DC to induce protective anti-tumor immunity [24]. In addition, enhanced local and systemic anti-tumor effects have been demonstrated when AdV transduced DC expressing cytokine genes have been injected intratumorally [22]. AdV have been utilized to transduce DC because they efficiently induce strong heterologous gene expression in these cells [24,25]. Prototypical vectors have now been extensively used in a variety of contexts [24,25].

CCL21 is a CC chemokine that belongs to a family of proteins involved in leukocyte chemotaxis and activation. Expressed in high endothelial venules and in T cell zones of spleen and lymph nodes, CCL21 exerts potent attraction of naïve T cells and mature DC promoting their co-localization in secondary lymphoid organs and cognate T cell activation [26]. We previously reported the potent anti-tumor properties of CCL21 in murine cancer models [27-29]. CCL21 has also shown anti-angiogenic activities in mice, thus strengthening its immunotherapeutic potential in cancer [30,31].

In our trial, DC will be transduced *ex vivo *with a replication incompetent adenovirus (by virtue of critical total deletions [E1 and partial deletion of E3 regions] in the adenoviral genome) expressing the CCL21 gene. Because autologous DC are transduced *ex vivo *and cells are sedimented, cultured, and extensively washed prior to injection into patients, this approach will contain the adenovirus entirely within the DC population so that it is unable to reproduce or infect adjacent cells. To date, no generation of replication competent adenovirus (RCA) has been detected following *in vivo *vector delivery [25]. In addition, no proviral integration or gene transfer to the gonads has been reported with the use of these vectors [25].

We have hypothesized that intratumoral injection of CCL21-gene modified DC (CCL21-DC) will stimulate specific immune responses without excluding patients on the basis of HLA phenotype or absence of a particular tumor antigen. CCL21-DC will have access to the entire repertoire of tumor antigens *in situ*. In addition, the CCL21-DC will exploit the professional APC as a vehicle for cytokine delivery, capitalizing on the capacity of CCL21 to attract both endogenous host DC and T lymphocytes to the tumor site to restore local immune reactivity. Here we report 1) an effective method for generation of clinical grade CCL21-DC from cryopreserved mononuclear cells (MNC) and 2) CCL21-DC are biologically active, secreting functional CCL21 capable of inducing chemotaxis *in vitro*. Intratumoral administration of clinical grade CCL21-transduced DC will be evaluated in a phase I clinical trial for late stage Non-Small Cell Lung Cancer. This therapeutic strategy is hypothesized to restore tumor antigen presentation and anti-tumor effector responses, by recruiting APC, T cells, and NK cells due to the chemotactic effect of CCL21 at the tumor site [27,28].

## Methods

### Generation of clinical grade AdCCL21

The CCL21 adenoviral construct (AdCCL21), lot# LO6042006, was manufactured for clinical use by the Biopharmaceutical Development Program at SAIC-Frederick (Frederick, MD) under FDA good manufacturing practice (GMP) standards. AdCCL21 is an E1-deleted replication-deficient serotype 5 adenoviral vector encoding the full-length cDNA for human CCL21 driven by the CMV promoter, as previously described [29]. AdCCL21 was originally constructed in our laboratory as follows: A 491 bp insert containing human CCL21 coding sequence 50–463, was amplified from human lymphocyte cDNA using the following primers: forward 5'-CTTGCAGCTGCCCACCTCAC (nucleotides 1–20) and reverse 5'-TCTCCAGGGCTCCAGGCTGC-3 (nucleotides 491-472). The CCL21 fragment was cloned into pAC-CMVpLpA to generate pAC-CMV-CCL21pA. This plasmid was co-transfected with pJM-17, which contains the E1-deleted Ad-genome, into 293 cells to yield recombinant E1-deleted adenovirus encoding CCL21 following homologous recombination. Clones of AdCCL21 were plaque-purified and viral seed stocks generated in 293 cells, followed by Cesium Chloride purification, dialysis, and storage at -80°C as a glycerol stock (10% volume/volume). The titer of each viral stock was routinely 10^11^-10^12 ^plaque-forming units (pfu) by plaque assay on 293 cells. Contamination with wild type virus or replication competent recombinant virus (RCA) was assessed by plaque-forming assay using the non-permissive A549 lung cancer cell line in parallel with the permissive 293-cell line. Only lots with 10^-4 ^pfu/ml were utilized for GMP amplification. GMP AdCCL21 was quantified by absorbance at 260 nm for particle number and by plaque assay on 293 cells for pfu. Each lot of virus was tested for presence of bacteria, fungal, mycoplasma and viral contamination, as well as for endotoxin contamination and RCA. Specifically viral stocks demonstrating > 1 RCA PFU/10^9 ^AdCCL21 PFU were discarded. An empty vector adenovirus identical to AdCCL21 but lacking the CCL21 insert (AdCV) was used as a control where indicated.

### Generation of human monocyte-DC

Peripheral blood mononuclear cells (PBMC) were obtained from leukocyte-enriched buffy coat (leukapheresis, LK) from healthy donors (UCLA Blood Bank) under the approval of UCLA Institute Review Board (IRB). Informed consent was obtained from each donor. PBMC were separated by density gradient centrifugation with Ficoll-Paque™ Plus (Amersham Biosciences, Uppsala, Sweeden). The light density mononuclear cell fraction from the 42.5–50% interface was recovered, washed three times and stored at -80°C in RPMI-1640 (Mediatech Inc., Herdon, VA) containing 20% AB serum (Gemini Bio-Products, Woodlands, CA) and 10% DMSO (Fisher Scientific, Fair Lawn, New Jersey). DC were cryopreserved up to one month without showing significant changes in viability after thawing (data not shown). Cryopreserved MNC were quickly thawed in a water bath, resuspended at 2 × 10^6 ^cells/ml in serum-free RPMI supplemented with 20 mM HEPES buffer (Mediatech Inc.), 100 units/ml Penicillin-Streptomycin (Invitrogen, Grand Island, NY), and 2 mM L-glutamine (Invitrogen) and allowed to adhere to tissue culture flasks for 2 h at 37°C. After incubation, non-adherent cells were discarded or utilized to generate autologous T cells for the Tetanus Toxoid (TT) assay (described below). Because DC are loosely adherent cells, they were harvested by gentle tapping of the flasks and pipetting. For optimal cell recovery, the flasks were washed twice in serum-free medium [29]. These cells were cultured for 6 days in complete RPMI with 5% human AB serum, 800 U/ml GM-CSF (Peprotech Inc., Rocky Hill, NJ), and 400 U/ml IL-4 (Peprotech). Although laboratory grade GM-CSF and IL-4 from Preprotech was utilized for these pre-clinical studies, in the phase I clinical trial, clinical grade GM-CSF and IL-4 cytokines will be used in accordance with GMP standards and in compliance with FDA requirements.

### Adenoviral transduction of DC

On day 6 of culture, monocyte-derived DC were harvested and cell viability was determined by Trypan Blue (Mediatech Inc.) exclusion while the viral vectors thawed on ice. Cells were equilibrated to room temperature (RT) for 15 minutes prior to mock transduction with serum-free culture medium (DC), or transduction with AdCCL21 (CCL21-DC) or AdCV (CV-DC) where indicated at 1167 viral particles (VP)/cell, equivalent to 100:1 multiplicity of infection (MOI). Transduction was carried out in serum free medium by centrifugation [33] using a temperature controlled microcentrifuge (Eppendorf, Fisher Scientific) for 2 h at 2000 × g to allow transduction to occur at RT or 37°C [29]. To test whether an immune adjuvant can improve DC function, 10 μg/ml clinical grade keyhole limpet hemocyanin (KLH) (BCI-Immunoactivator, Intracel Resources, Frederick, MD) was added to 0, 30, or 100% of DC, CV-DC, or CCL21-DC prior to centrifugation. After transduction, cells were washed three times and resuspended in RPMI containing 5% AB serum. Cells were counted and viability was determined by Trypan Blue exclusion within two hours following transduction. We expect a maximum delay of two hours prior to intratumoral administration due to the safety testing required to fulfill lot release criteria. Cell viability at two hours following adenoviral transduction was confirmed by annexin-V (AV) and propidium iodide (PI) staining using the AV fluorescein isothiocyanin (FITC) kit (Biosource International, Camarillo, CA) in accordance to the manufacturer's instructions. DC, CCL21-DC, and CV-DC were seeded at several densities and cultured over a 72 h time course to assess CCL21 production/cell.

### Immunophenotypic analysis by flow cytometry

DC, CCL21-DC, and CV-DC were characterized by flow cytometry after transduction using the following panel of monoclonal antibodies: HLA-DR-FITC, CD40-phycoerytrin (PE), CD54-PE, CD80-PE, CD86-PE, and CCR7-PE (BD Biosciences Pharmingen, San Diego, CA), CD83-FITC (Coulter Immunology, Hialeah, FL), and appropriate isotype controls (BD Biosciences, La Jolla, CA). Ten thousand events were acquired within a pre-set DC gate using a LSR flow cytometer (BD Biosciences, San Jose, CA) and analyzed using CELLQuest software (BD Biosciences, San Jose, CA).

To measure dextran (DX) uptake, DC and CCL21-DC were incubated with FITC-conjugated dextran (DX-FITC) (Sigma, San Louis, MO, 1 mg/ml) in RPMI with 10% AB serum for 20 minutes at 37°C. A parallel protocol was also carried out at 4°C to assess non-specific FITC signals. After incubation, cells were washed twice in FACS buffer (PBS (Mediatech Inc.) containing 2% FBS (Gemini) and incubated with anti-CD86-PE or isotype control for 30–45 minutes at 4°C. After washing DC were fixed in 400 μl 1% paraformaldehyde (Electron Microscopy Science, Ft. Washington, PA) and analyzed by flow cytometry. Ten thousand events were acquired within a pre-set DC gate using a LSR flow cytometer, as described above.

To evaluate expression of CCR7, T2 cells and day 6 immature DC (IDC) were incubated with CCR7-PE, appropriate rat isotype IgG isotype control, or FACS buffer alone for 20 minutes at RT. Cells after washing in FACS buffer were analyzed using a LSR flow cytometer (BD Biosciences, San Jose, CA) as described.

### Analysis of transduction efficiency

To evaluate the efficiency of gene delivery, we utilized the reporter gene green fluorescent protein (GFP). Briefly, DC were transduced with culture medium (DC) or with a replication deficient adenoviral vector carrying the gene encoding GFP (Ad-CMV-GFP, Vector Biolabs, Philadelphia, PA), driven by the CMV immediate-early promoter (GFP-DC). Transduction was carried out in serum-free medium at 1167 VP/cell by the centrifugation method at RT, as described above. Cells were cultured for 24 or 48 h prior to analysis of GFP expression. To identify the dendritic cell population, both DC and GFP-DC were incubated with CD86 PE antibody or isotype control in FACS buffer for 20 minutes at RT. Cells were then analyzed for GFP/CD86 expression by flow cytometry with the LSR flow cytometer, as described above. Five thousand events were collected within a pre-set DC gate.

### CCL21 ELISA

CCL21 protein concentration in the supernatant of DC, CV-DC and CCL21-DC was determined by CCL21-DuoSet specific ELISA (R&D Systems, Minneapolis, MN) following manufacturer's instructions. Briefly, monoclonal antibody directed against CCL21 (2 μg/ml) was allowed to adsorb to a 96-well plate (Costar, Cambridge, MA) overnight. After three washes in PBS containing 0.05% Tween-20 (PBS-T), wells were incubated for 1 h at RT with PBS containing 10% bovine serum albumin (BSA) (Sigma) to block non-specific binding. On the following day, CCL21 standard or sample supernatant were added to the wells and incubated for 2 h at RT. Unbound antigen was removed by washing with PBS-T and the plate was incubated with biotinylated anti-CCL21 antibody for 2 h at RT, washed and incubated with streptavidin-conjugated HRP (1:200) for 20 minutes in the dark. After washing to remove unbound reagents, the plate was incubated with substrate for up to 30 minutes in the dark. The reaction was stopped by adding 1 M sulfuric acid to the wells and the optical density at 450 nm was determined using a microplate reader (Benchmark, Bio-Rad, Hercules, CA). The sensitivity of this CCL21 assay was 62.5 pg/ml.

### IL-12p_70_, IP-10, and MIG ELISA

To induce IL-12 production, 2.0 × 10^5 ^DC, CV-DC, or CCL21-DC were primed with IFN-γ (50 ng/ml, Peprotech Inc.) for 2 h and then stimulated with LPS (1 μg/ml, Sigma), for 48 h. To induce IP-10 and MIG production, DC, CV-DC, or CCL21-DC were stimulated with LPS (1 μg/ml) for 24 h. IL-12p_70 _concentration in the cell supernatant following 48 h stimulation was determined with the Ready-set-go! ELISA (eBioscience, San Diego, CA) following manufacturer's instructions. The sensitivity of the IL-12p_70 _ELISA was 4 pg/ml. IP-10/CXCL10 and MIG/CXCL9 production were determined 48 h after stimulation with specific ELISA kits (BD Bioscience), following manufacturer's instructions. The sensitivity of the IP-10/CXCL10 and MIG/CXCL9 ELISAs were 8 pg/ml and 30 pg/ml, respectively.

### Allogeneic mixed lymphocyte reaction and autologous TT presentation assays

To assess the ability of CCL21-DC to induce proliferation of allogeneic T cells and to evaluate the impact of KLH on their APC function, we utilized allogeneic T cells as responder cells in a mixed lymphocyte reaction (MLR) assay. Briefly, MNC were obtained from PBMC by density gradient separation as described above. After incubation for 2 h in tissue culture flasks, non-adherent cells were isolated and plated into a 96-well round-bottom plate at 2.0 × 10^5 ^cells/well. KLH (10 μg/ml) was added to 0%, 30%, or 100% of DC, CV-DC, or CCL21-DC prior to centrufugation, as described in the paragraph of transduction. For preparation of APC, 0% pulsed or 30–100% pulsed DC, CV-DC, or CCL21-DC were pre-treated with mitomycin C (50 μg/ml, Sigma, San Louis, MO) for 30 minutes in a 37°C water bath. After three washes all DC samples were resuspended in RPMI medium containing 10% AB serum and mixed with responder cells at DC to responder cell ratios of 1:2, 1:5, or 1:10. T cell proliferation was assessed with the BrdU Cell Proliferation ELISA kit (Roche Applied Science, Indianapolis, IN) following manufacturer's instructions. The incorporation of 5'bromo-2'-deoxyuridine (BrdU) was evaluated by measuring the optical density (OD) of the replicating responder T cells (405 nm) after 5 days incubation at 37°C, 5% CO_2_.

To assess autologous antigen presentation by adenoviral-transduced DC we employed a standard TT presentation assay as previously described with some modifications [32]. Autologous T-cells were generated from non-adherent MNC during preparation of DC. Briefly, non-adherent MNC were cultured at 1.0 × 10^6 ^cells/ml in the presence of IL-2 (15 U/ml, Dr. James Economou, UCLA, Los Angeles, CA) for 6 days. KLH (10 μg/ml) was added to 0, 30. or 100% of DC, CV-DC, and CCL21-DC previously treated with mitomycin C. Autologous T cells were then admixed to the DC at 10:1 ratio. TT (2 μg/ml, Calbiochem, San Diego, CA), BSA (Genzyme, Cambridge, MA), or culture medium alone were added.

T-cell proliferation was assessed by BrdU Cell Proliferation assay after 5 days incubation at 37°C, 5% CO_2_, as described above while effector function was evaluated by measuring IFN-γ production, after 48 h culture by specific ELISA (eBioscience, San Diego, CA), following manufacturer's instructions. The sensitivity of the IFN-γ ELISA was 4 pg/ml.

### Chemotaxis Assay

Chemotaxis assays were performed with T2 cells, a T/B hybridoma that lacks HLA class II antigen but expresses HLA.A2 molecules, commonly used to assess specific cytotoxic T lymphocyte responses. We evaluated the chemotactic activity of supernatant from DC, CV-DC, and CCL21-DC as described previously [29]. Briefly, T2 cells (provided by Peter Cresswell, Yale University School of Medicine, New Haven, CT) were harvested, counted, and resuspended in serum-free RPMI medium at 2 × 10^6 ^cells/ml. 100 μl were loaded into the upper chamber of a standard 24-well plate fitted with 3 μm polycarbonate membrane inserts (Corning Life Science, Corning NY). 400 μl of supernatant from DC, CV-DC, or CCL21-DC was added to the lower chamber of each well. 600 ng/ml recombinant CCL21 (ED_50_: 200–600 ng/ml) and 10% AB serum in RPMI were used as positive and negative controls, respectively. For total migration, T2 cells were added without the membrane barrier. After incubation for 2 and 1/2 h, migrated cells were recovered from the lower chamber and quantified by flow cytometry based upon the number of events per minute collected in a pre-set T2 cell gate. To evaluate the specific chemotactic activity of CCL21 produced by CCL21-DC, supernatant from DC, CV-DC, CCL21-DC or 600 ng/ml recombinant CCL21 protein were exposed to neutralizing concentrations of anti-CCL21 antibody (R&D Systems, ND_50_: 1–4 μg/50 ng rCCL21) or isotype control (ChromPure Goat IgG, Jackson ImmunoResearch Laboratories, West Grove, PA) prior to loading into the transwell plate and all subsequent steps were carried out as described above.

### Statistical Analysis

The unpaired two-tailed Student's t test and the one-way ANOVA for multiple groups were used to compare differences among DC, CV-DC, and CCL21-DC. p values = 0.05 were considered significant.

## Results

### Generation of DC from cryopreserved human MNC

Our phase I clinical trial entailing two sequential intratumoral injections of gene-modified DC will require readily accessible stocks of autologous MNC. This prompted us to examine cryopreserved MNC as an optimal source of cells that would be available in a timely manner. To assess the feasibility of this approach, a series of pilot experiments was performed using leukapheresis specimens from healthy volunteers. Seventeen MNC samples were evaluated after cryopreservation in 10% DMSO and 20% AB serum in RPMI medium after Ficoll gradient separation. The *in vitro *pre-clinical experiments were performed with MNC that were cryopreserved at -80°C for up to one month. Although this storage time is beyond the cryopreservation time that will be utilized in the clinical trial, we anticipate initiation of DC culture one week following the cryopreservation of MNC. Under our experimental conditions, cryopreservation of MNC up to 30 days did not result in significant changes in cell viability and yield (data not shown). As shown in Table 1, the yield for MNC before cryopreservation averaged 1 × 10^9^± 0.7 cells in 17 experiments. The MNC recovery after thawing averaged 0.66 × 10^9 ^± 0.4 cells with 92.9 ± 4.3% viability, as determined by Trypan Blue exclusion. DC were obtained from cryopreserved MNC by culture of adherent monocytes with laboratory grade GM-CSF and IL-4, as described in *Materials and Methods*. After 6 days in culture, the DC yield from 0.66 × 10^9 ^MNC averaged 21.3 × 10^6 ^± 11 (n = 17) and exhibited an average viability of 95.8 ± 1.7% in 16 independent experiments (Table 1). These results are in agreement with our previous study [29]. Immunophenotypic analysis by flow cytometry using a panel of cell surface markers described previously [29] confirmed that DC generated in this manner maintained an immature phenotype (data not shown).

**Table 1:** Generation of human DC and adenoviral CCL21 transduced DC from cryopreserved MNC.

	**MNC** **Yield** ** (BC)**	**MNC** **Recovery** ** (AC)**	**MNC ** **Viability** ** (AC)**	**DC** **Yield** ** (BT)**	**DC** Viability* (BT)	**AdCCL21** **Recovery** ** (AT)**	**AdCCL21** **Viability** ** (AT)**
N = 17	1 × 10^9^	1 × 10^9^	%	1 × 10^6^	%	%	%
	
	1.0 ± 0.7	0.66 ± 0.4	92.9 ± 4.3	21.3 ± 11	95.8 ± 1.7	65.9 ± 7.4	88 ± 13%

* N = 16

DC were generated from cryopreserved MNC obtained from leukapheresis (LK) samples under standard culture conditions (6 days culture of adherent MNC in GM-CSF and IL-4), as described in *Materials and Methods*. A summary of the yield, recovery, and viability of MNC is reported before cryopreservation (BC), after cryopreservation (AC), before adenoviral transduction (BT), and after adenoviral transduction (AT) with AdCCL21 at room temperature. The values express the average ± SD of at least 16 independent experiments.

### Adenoviral transduction with clinical grade AdCCL21 generates viable biologically active DC

On day 6 of culture DC were transduced with clinical grade AdCCL21, AdCV, or mock transduced with culture medium alone, as described [29,32]. To assess the effect of temperature on DC viability and CCL21 production, preliminary transduction experiments (n = 4) were carried out in parallel at RT and 37°C. Cell viability and yield were determined by Trypan Blue exclusion. Transduction at 37°C and RT resulted in equivalent DC recovery (50.7 ± 14% versus 59 ± 0.03%, p > 0.05), but significantly diminished cell viability (75 ± 0.2% versus 93.6 ± 0.02%, p = 0.05), respectively (Table 2). Accordingly, all subsequent transductions were performed at RT. Cell recovery, following adenoviral transduction at RT, was 65.9 ± 7.4% of the initial DC yield in 17 independent experiments, and the overall viability of CCL21-DC averaged 88 ± 13% (n = 16), as determined by Trypan Blue exclusion (Table 1). Analysis of apoptosis by AV and PI confirmed these observations, showing an average viability of 89 ± 5% at two hours following adenoviral transduction (n = 3, data not shown).

**Table 2:** Impact of temperature on AdCCL21 transduction of DC.

**AdCCL21-DC**							
Recovery		**Viability**		**CCL21**			
37°C	RT	37°C	RT	37°C		37°C	

				ng/1 × 10^6^	ng/1 × 10^6^	ng/1 × 10^6^	Ng/1 × 10^6^
**%**				24 h	48	24 h	48

50.7 ± 14	59 ± 0.03	75 ± 0.2	*93.6 ± 0.02	1.1 ± 0.7	1.9 ± 1.3	2.9 ± 2.1	3.7 ± 2.5

• p = 0.05

In preliminary experiments (n = 4), the impact of temperature on adenoviral transduction of human DC was evaluated. Cell recovery, viability by trypan blue exclusion, and CCL21 production are shown at 37°C and RT.

Transgene expression in CCL21-DC was assessed by measuring CCL21 production in the supernatant of DC, CV-DC, or CCL21-DC seeded at several densities over a 72 h time course (Table 3). Production of CCL21 averaged 3.5 ± 3.4 ng/5 × 10^6 ^cells at 24 h (n = 6), 4.4 ± 2.9 ng/5 × 10^6 ^cells at 48 h (n = 7), and 6.7 ± 1.5 ng/4 × 10^6 ^cells at 72 h (n = 4). Maximal CCL21 production was obtained with 1 × 10^7 ^CCL21-DC (7.5 ± 2.4 ng at 24 h and 8.7 ± 1.1 ng at 48 h). Neither DC nor CV-DC generated detectable CCL21 (Table 3). CCL21 protein levels appeared to be affected by temperature during transduction as demonstrated by a trend toward higher CCL21 production when DC were transduced at RT compared to 37°C (1.1 ± 0.7 ng versus 2.9 ± 2.1 ng at 24 h, and 1.9 ± 1.3 ng versus 3.7 ± 2.5 ng at 48 h, respectively; p > 0.05, Table 2).

**Table 3:** CCL21 production.

**CCL21 in DC supernatant** ** [ng]/cell number**													
**24 h**						**48 h**						**72 h**	
**DC**	CCL21-DC				CV-DC	DC	CCL21-DC				CV-DC	DC	CCL21-DC
Cell number: ×10^6^						Cell number: ×10^6^							
1	1	2.5	5	10	1	1	1	2.5	5	10	1	1	4
n = 16	n = 16	n = 9	n = 6	n = 2	n = 6	n = 12	n = 10	n = 7	n = 7	n = 2	n = 6	n = 4	n = 4
ng/cell number						ng/cell number						ng/cell number	

*0.0	2.3 ± 1.9	3.8 ± 2.8	3.5 ± 3.4	7.5 ± 2.4	*0.0	*0.0	3.7 ± 2.4	5.2 ± 2.5	4.4 ± 2.9	8.7 ± 1.1	*0.0	*0.0	6.7 ± 1.5

* Below detection

The capacity of DC to secrete CCL21 was evaluated by ELISA. Mock-transduced DC (DC), CV-DC and CCL21-DC were cultured at the concentrations indicated in 1 ml culture medium. The supernatant was analyzed after 24, 48, and 72 h cultures. The concentration of CCL21 is expressed in ng per cell number. The values represent the average ± SD of different independent experiments, as indicated.

### Efficiency of adenoviral transduction

**Figure 1:**
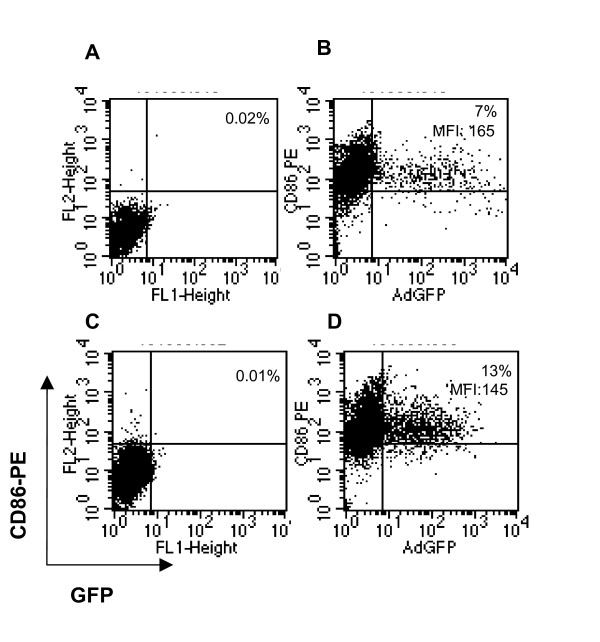
**Susceptibility of human DC to adenoviral transduction**. DC on day 6 of culture were transduced with an adenovirus encoding the marker gene GFP under the same conditions utilized to transduce CCL21-DC. After transduction, DC were cultured up to 48 h. GFP expression was analyzed by flow cytometry at 24 h (**A, B**) and 48 h culture (**C, D**), as described in *Materials and Methods*. A population of GFP^+ ^CD86^+ ^DC at 24 h (**B**) and 48 h (**D**) is shown. Staining with isotype control is indicated in **A **and **C**. The percentage of GFP^+ ^CD86^+ ^DC is indicated in the upper right corner, and MFI is also reported for D and C. The density plots for one representative experiment of four are shown.

The initial attachment of adenovirus to the majority of human cell types is mediated by its fiber capsid protein, which binds to the high-affinity Coxsackie-Adenovirus Receptor (CAR) [25]. MHC molecules may also serve as receptors for the fiber capsid protein [25]. Alternatively adenovirus may bind to cells of hematopoietic origin through αMβ_2 _integrins [25]. Subsequent internalization is mediated by αVβ_3 _or αVβ_5 _integrins. While immature DC as well as adenovirus infected-DC or DC matured with LPS, CD40L, TNF-α, monocyte-conditioned medium (MCM) and poly (I-C) all lack CAR and αVβ_3 _integrins, αMβ_2 _and αVβ_5 _integrins are expressed at higher levels in immature DC compared to mature DC [25]. Thus, viral transduction of human monocyte-derived DC requires immature cells and often a high MOI. Adenoviral transduction efficiency was assessed by analyzing expression of a GFP reporter gene measured by flow cytometry. DC were transduced with an adenovirus expressing GFP under similar conditions used for transduction with AdCCL-21. GFP production measured by flow cytometry revealed 7 and 13% of DC expressed GFP following 24 and 48 hours from transduction, respectively. The density plots are shown in Figure 1.

### Immature DC phenotype is maintained after AdCCL-21 transduction

**Figure 2:**
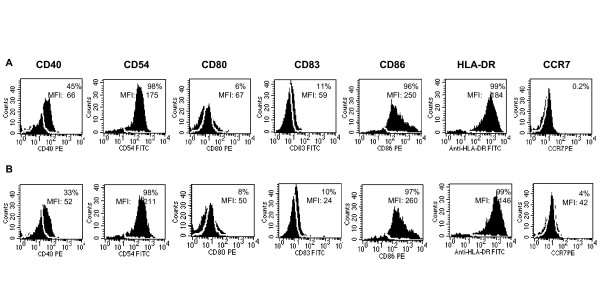
**DC phenotype is conserved after transduction with AdCCL21**. DC (**A**), and CCL21-DC (**B**), were analyzed for expression of the following surface markers: CD40, CD54, CD80, CD83, CD86, HLA-DR and CCR7, as indicated. Histograms show the expression of each surface molecule individually. White lines contour the isotype control. The percentage of positive cells and MFI are indicated. A representative experiment of at least three is shown.

**Figure 3:**
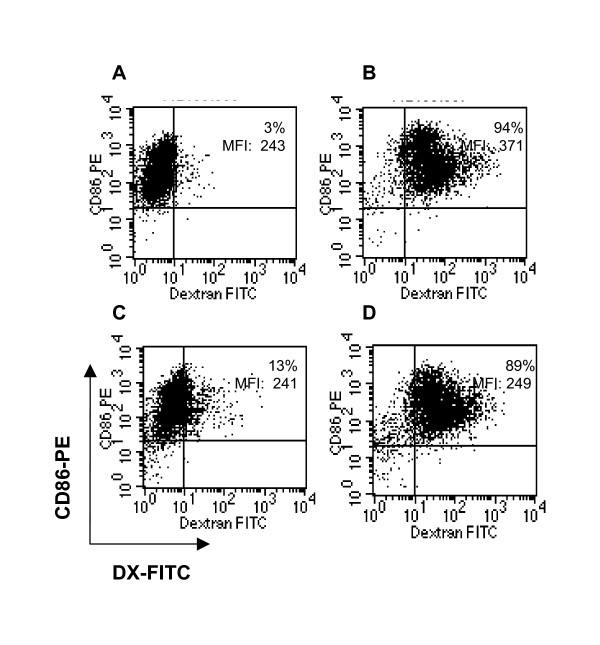
**Dextran uptake by CCL21-DC**. DC (**A **and **B**) and CCL21-DC (**C **and **D**) were incubated with DX-FITC at 4°C and 37°C for 15 minutes, as described in *Materials and Methods*. DX expression was analyzed in CD86^+ ^DC. A population of DX^+ ^CD86^+ ^obtained at 4°C and at 37°C is shown. Values indicate the percentage and MFI of DX^+ ^CD86^+^. Density plots of a representative experiment of three are shown.

A large body of evidence has emphasized the importance of DC differentiation in the outcome of adoptive transfer in cancer patients [10-12]. The effectiveness of DC based immunotherapy to control human cancer is controversial. It is especially debated whether DC that infiltrate a tumor result in tumor-specific tolerance rather than immunization [34]. Immature DC appear to be more efficient than mature DC at antigen internalization and processing and more effectively augment tumor-specific immune responses [35]. In our current studies, DC conserved an immature phenotype after transduction with AdCCL21 (Figure 2). There were no significant differences in cell surface expression of co-stimulatory molecules or maturation markers (CD40, CD54, CD80, CD83, CD86, and HLA-DR) between DC (A) and CCL21-DC (B) with the exception of a modest up-regulation of the CCL21 receptor (CCR7), as we previously observed [29]. CCL21-DC also retained the ability to internalize dextran comparable to that of non-transduced DC (Figure 3B and 3D). These experiments confirmed the immature phenotype of CCL21-DC as well as their capacity to endocytose antigen, which has been suggested to be fundamental in the initiation of tumor-specific immune responses [8,9].

### CCL21-DC are effective antigen presenting cells

**Figure 4:**
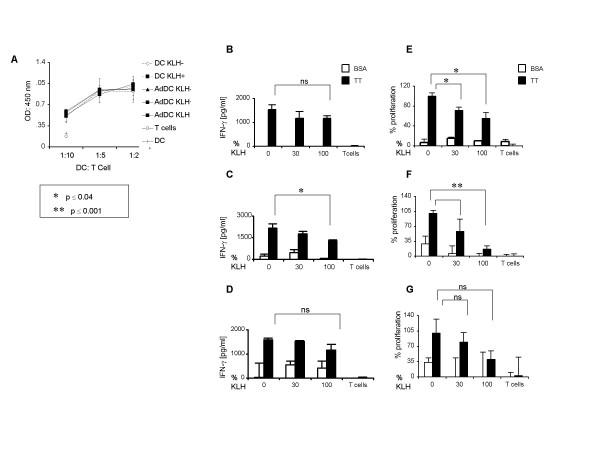
**CCL21-DC are efficient APC in the absence of KLH**. (A) Allogeneic MLR: Briefly, culture medium mock-transduced DC (DC) and CCL21-DC (AdDC) were analyzed for their ability to induce allogeneic T cell proliferation. The effect of pulsing 100% (DC KLH 100 and AdDC KLH 100) or 30% of cells (AdDC KLH 30) with 10 μg/ml KLH prior to initiating the MLR assay was assessed. After pre-treatment with mitomycin C and KLH, where indicated, DC and CCL21-DC were mixed with allogeneic T cells at several ratios, as shown. Cell proliferation was assessed by BrdU incorporation following 5 days incubation at 37°C. One representative experiment of three is shown. **(B-G) Autologous T cell proliferation assay: **T cells were cultured for 6 days with IL-2 (15 U/ml) prior to the TT presentation assay. After mitomycin-C treatment and KLH pulsing where indicated, DC (**B, E**), CCL21-DC (**C, F**), or CV-DC (**D, G**) were mixed at 1:10 ratio to autologous T cells in the presence of TT (2 μg/ml) or BSA (2 μg/ml). In (**B-D**), IFN-γ production was measured in the cell supernatant collected after 48 hours of cell proliferation. The measured values of IFN-γ detected by ELISA were within the linear portion of the IFN-γ concentration curve. The concentration of IFN-γ is expressed in pg/ml with basal IFN-γ production subtracted. In (**E-G**), T cell proliferation in response to TT and BSA was measured by BrdU incorporation following 5 days co-culture at 37°C and is expressed as a percentage of proliferating cells. One representative experiment of at least three is shown. P value ≤ 0.05 was considered significant.

Human DC contain suppressive subsets [36,37] raising the concern that *ex vivo *transduction may select for these suppressive cells and their re-introduction may induce local immunosuppression or ineffective recruitment of T cells. Keyhole limpet hemocyanin (KLH) is an immune adjuvant and hapten carrier which has been used in combination with other immunotherapy strategies. Based on this function, in the clinical protocol, KLH was planned to be added as an immune adjuvant to our CCL21 gene modified DC. However, KLH conjugate vaccines have shown inconsistent results in clinical trials [38-40]. To address these concerns and conflicting data on its immune enhancing function, the biological activity of CCL21-DC was examined, and the impact of KLH pulsing was assessed in both allogeneic and autologous settings *in vitro*. Because KLH has resulted in variable effects based on the number of pulsed cells, the impact of KLH on DC APC capacity was tested by incubating 100%, 30%, and 0% of DC, CV-DC, and CCL21-DC with KLH. CCL21-DC stimulated T cell proliferation comparable to DC in the allogeneic MLR in the presence or absence of KLH (Figure 4A). However, in autologous TT presentation assays when KLH was added to CCL21-DC, a significant reduction in IFN-γ production was noted (Figure 4C). In contrast, it did not significantly affect DC (Figure 4B) or CV-DC (Figure 4D). Similarly, we observed impaired proliferation of autologous T cells (Figure 4E–4F) when either 30 or 100% of DC (Figure 4E) or CCL21-DC (Figure 4F) were pulsed with KLH prior to combining with T cells. In this setting, CV-DC did not show a significant effect in the induction of autologous T cell proliferation to TT, with or without KLH (Figure 4G). The background levels of IFN-γ for DC, CV-DC, and CCL21-DC expressed as range and (mean) were 0 (0), 2242.2–2532 (2523), and 44.2–460.4 (239.5) pg/ml, respectively. The DC group had IFN-γ levels under the detection limit of the ELISA assay.

### CCL21-DC maintain ability to secrete immune activating cytokines and chemokines

**Figure 5:**
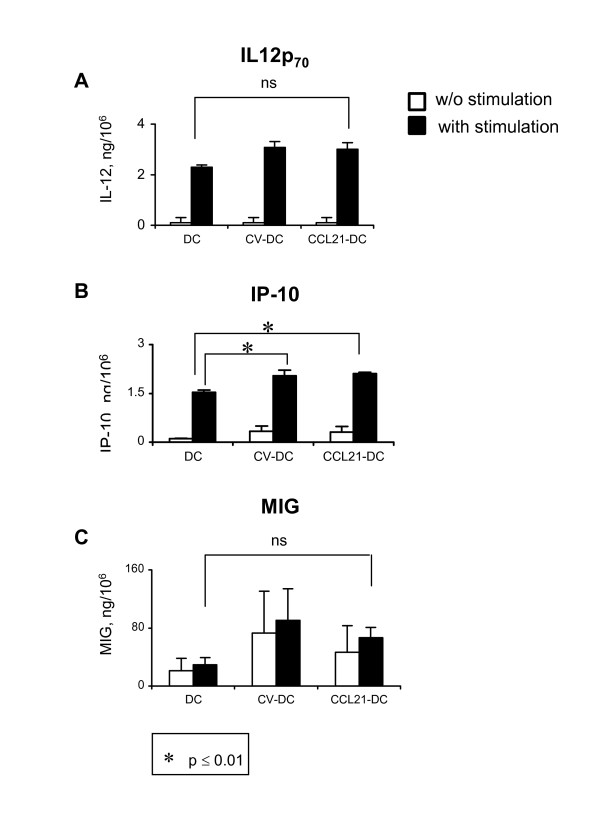
**CCL21-DC secrete immunostimulatory cytokines**. Human monocyte-derived DC were transduced with culture medium, AdCCL21 (1167 VP/cell), or AdCV by the centrifugation method. 2.0 × 10^5 ^DC, CCL21-DC, or CV-DC were seeded into a 48 well plate in 1 ml of culture medium in the presence or absence of the indicated stimuli. For IL-12 assays, cells were stimulated with IFN-γ + LPS, and for IP-10 and MIG assays cells were stimulated with LPS only. The supernatants from stimulated and unstimulated DC were harvested after 48 h culture. IL-12p_70 _**(A**), IP-10 **(B)**, and MIG **(C) **were measured by ELISA. Values refer to cytokine concentration expressed in ng/million cells. One representative experiment of four is shown.

We have previously reported that transduction of DC with laboratory-grade AdCCL21 vector did not impair the secretion of IL-12 [29], a critical cytokine for induction of Th1-mediated anti-tumor immune responses. To further investigate the immunological properties of CCL21-DC, IL-12 production was analyzed from these cells upon stimulation with IFN-γ and LPS. CCL21-DC and CV-DC maintained the ability to become activated upon stimulation and secrete IL-12 in comparable levels to non-transduced, control DC (Figure 5A).

Given that IP-10/CXCL10 and MIG/CXCL9 are important for CCL21-DC mediated tumor regression in murine studies [27,28], we examined whether AdCCL21 transduction increased the expression of these chemoattractants in human DC. Our data demonstrate that upon stimulation with IFN-γ and LPS, CV-DC and CCL21-DC secreted significantly higher levels of IP-10/CXCL10 compared to non-transduced DC, suggesting an adenoviral-mediated effect on IP-10 production (Figure 5B). Furthermore, CCL21-DC and CV-DC maintained the ability to become activated with stimulation and secrete IP-10/CXCL10 in comparable amounts to that of non-transduced DC (Figure 5B).

With respect to MIG/CXCL9 production, adenoviral transduction does not impair the ability of DC to secrete MIG (Figure 5C). CCL21-DC and CV-DC maintained MIG/CXCL9 levels comparable to that of control DC in both stimulated and non-stimulated conditions (Figure 5C). Altogether the *in vitro *data in human DC support our previous findings from murine models in which intratumoral injection of CCL21-DC led to the increased production of IFN-inducible chemokines and cytokines associated with tumor regression [27,28].

### Supernatants from CCL21-DC stimulate chemotaxis of T2 cells

**Figure 6:**
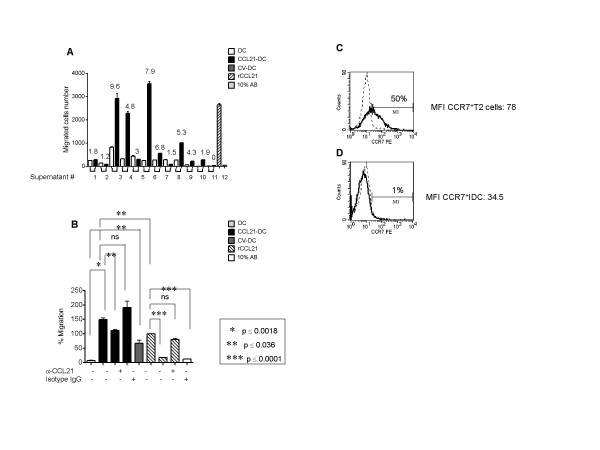
**Supernatants from CCL21-DC stimulate chemotaxis of T2 cells**. **(A) **The supernatants from DC, CCL21-DC and CV-DC from different individuals (n = 12) were utilized in a standard chemotaxis assay to assess their ability to induce chemotaxis of T2 cells. Briefly 2.0 × 10^5 ^T2 cells were loaded in the upper chamber of a 24-well transwell apparatus and allowed to migrate through a 3 μm insert for 2 and 1/2 h at 37°C. In **A**, the lower chambers of the transwell had been loaded with the supernatant from DC or CCL21-DC (supernatant #1–11) from the same individual, or DC or CV-DC (#12) from the same individual, as indicated. Recombinant CCL21 (600 ng/ml) and 10% AB medium were added to the wells as positive and negative controls, respectively. A summary of chemotaxis from 12 different samples is shown. The CCL21 protein concentration in ng/ml is indicated over each column. Migrated cells were analyzed by flow cytometry by counting the number of events per minute (expressed as migrated cell number) within a pre-determined T cell gate. One-way ANOVA p < 0.0001. In blocking experiments (**B**), the supernatants from CCL21-DC or recombinant CCL21 were pre-treated with a neutralizing concentration of anti-CCL21 antibody (1.5 μg/50 ng CCL21) or isotype immunoglobulin, prior loading in the lower chamber of the transwell apparatus. The supernatant from CV-DC and medium containing 10% AB were also assessed. To determine total T2 cell migration, T2 cells were loaded without inserts. A representative experiment of a total of seven is shown. The number of migrated cells was analyzed by flow cytometry as above, and is expressed as percentage of total T2 cell migration. Asterisks denote statistical significance (p < 0.05). The surface expression of CCR7 is analyzed by flow cytometry in T2 cells (**C**) and in immature DC (**D**) cultured for 6 days in GM-CSF and IL-4. Histograms are shown: dotted lines indicate isotype control, bold lines indicate marker expression. The percentage of positive cells and MFI are indicated. A representative experiment of two is shown.

Because we hypothesized that CCL21-DC would enhance chemoattraction of stimulatory T cells and improve the probability of cognate T cell interaction and cross-presentation, [29] we investigated the ability of CCL21-DC supernatant to elicit migration of T2 cells. Given the known experimental variability of using human CD4^+ ^and CD8^+ ^T cells purified from different individuals, T2 cells were used due to the known and consistent T2 cell CCR7 expression and the ability to reproducibly culture the cells *in vitro*. Supernatant containing as little as 4.8 ng/ml of CCL21 secreted by at least 2 × 10^6 ^CCL21-DC, was sufficient to induce a marked increase in chemotaxis of T2 cells (One-way ANOVA: p < 0.0001, Figure 6A) compared to supernatant from non-transduced DC, CV-DC, or diluent (10% AB) control (Figure 6A and 6B). Recombinant CCL21 (600 ng/ml) induced chemotaxis was almost completely abrogated by neutralizing concentrations of anti-CCL21 antibody but not by the isotype control antibody (Figure 6B). The chemotactic effect of the CCL21-DC supernatant was only partially suppressed by neutralizing concentrations of anti-CCL21 antibody, and continued to demonstrate pronounced induction of chemotaxis compared to DC (Figure 6B). As shown in figure 6B, in some experiments, the supernatant from DC transduced with AdCV induced significant induction of chemotaxis of T2 cells compared to DC alone. While this suggests a vector-mediated chemotactic effect, CCL21-DC consistently caused a greater degree of chemotaxis. The CV-DC supernatant induction of T2 cell chemotaxis was not affected by the addition of anti-CCL21 antibody (data not shown).

Because CCL21 chemotaxis implies expression of CCR7 on the effector cell surface, the expression of CCR7 in T2 cells was analyzed utilizing immature DC at day 6 of culture as a negative control. T2 cells expressed high levels of CCR7 (50%, Figure 6C) compared to immature DC (1%, Figure 6D).

## Discussion

In this report, we describe an effective method for generating human gene-modified DC for therapeutic use in a phase I clinical trial of advanced NSCLC. We have optimized a procedure for differentiation of human DC from cryopreserved MNC obtained by leukapheresis, followed by transduction with a clinical grade E1-deleted, replication-deficient adenoviral vector, encoding the CCL21 gene. Adenoviral transduction resulted in sufficient quantity of viable immature CCL21-DC for intratumoral administration. Autologous clinical grade CCL21-DC will be evaluated in a single center, non-randomized, dose escalation phase I clinical trial, for subjects with Stage IIIB and IV NSCLC. The proposed intratumoral injection of CCL21 gene modified DC is anticipated to promote co-localization of mature host DC and Th1 lymphocytes at primary lung tumor, and the use of the tumor as an *in vivo *source of antigens will presumably allow the induction of a specific immune response against a repertoire of tumor associated antigens (TAA).

We have previously reported our preclinical data on the use of intratumoral injection of CCL21-DC in murine models of established lung cancer resulting in tumor eradication by eliciting a systemic anti-tumor immune response that provided protection against subsequent challenge with the same tumor cell line [27,28]. Here we report that 1) human DC can be efficiently transduced *ex vivo *with clinical grade adenoviral vector expressing the CCL21 gene, 2) transduction of DC with AdCCL21 at RT improved cell viability over that observed after transduction at 37°C, 3) CCL21-DC demonstrate an immature DC phenotype with the ability to phagocytose and present antigens, 4) KLH inhibits the ability of CCL21-DC to induce T cell proliferation and cytokine production, 5) CCL21-DC maintain the ability to be activated and produce IL-12 and transduction does not impair DC generation of IP-10/CXCL10 and MIG/CXCL9, and 6) CCL21-DC secrete CCL21 protein within 24 h of transduction and demonstrate biological activity with induction of chemotaxis.

Given these *ex vivo *findings, CCL21-DC upon intra-tumoral injection/re-introduction into the patient may demonstrate *in vivo *ability to internalize and present tumor associated antigens to host effector cells eliciting a specific anti-tumoral response, as well as, having the biological activities specific to CCL21. In murine studies, CCL21-DC induced tumor regression, stimulated production of IFN-γ and the CXC chemokines MIG (CXCL9) and IP-10 (CXCL10) at the tumor site, and decreased local concentrations of immunosuppressive inflammatory mediators including IL-10, PGE_2_, and TGF-β [27,31]. These changes in cytokine expression were required for an effective CCL21-DC mediated anti-tumoral response [28]. *In vivo *depletion of MIG, IP-10, or IFN-γ individually or in combination reduced the anti-tumor efficacy of CCL21-DC [27].

The rationale for utilizing CCL21-DC as opposed to direct injection of AdCCL21 or recombinant CCL21 is derived from pre-clinical murine studies in which CCL21 administered as a recombinant protein showed anti-tumor properties only at very high doses [27,28]. Similarly, injection of the AdCCL21 vector directly into murine tumors was only effective at high pfu (greater than 10^7 ^VP in 3 sequential intratumoral injections) [27,28], which is not feasible for clinical delivery. Therefore, the DC component may be critical for clinical anti-tumor responses. DC may also shelter the adenoviral vector from the anti-viral humoral response of the patient [25]. In previous studies, direct immunization of mice with recombinant adenovirus resulted in induction of high titers of neutralizing antibodies and inhibition of CTL response in repeated inoculations [41]. However, DC harboring adenovirus 5, E1-deleted vectors induce low titers of neutralizing antibodies [25]. The capacity of mice immunized with CCL21-DC to reject a secondary challenge with tumor cells demonstrates that CCL21-DC effectively generate a systemic CTL response and enhance tumor immunogenicity [27,28]. Studies to reveal the molecular mechanism underlying improved response to CCL21-DC over direct intratumoral injection of the AdCCL21 viral vector itself or recombinant CCL21 are underway in our laboratory.

In the clinical trial, we expect a maximum delay of two hours prior to intratumoral administration of our final product (CCL21-DC) into the patient's primary lung cancer due to the required safety testing to fulfill lot release criteria. Our results revealed that the viability of CCL21-DC at two hours following adenoviral transduction by trypan blue exclusion (88 ± 13%) and confirmed by annexin V (AV) staining (89 ± 5%) will successfully meet the final lot release criteria for cell viability, which is currently defined to be > 70%.

CCL21-DC exhibited an immature DC phenotype and intact immune stimulatory capability. Although these DC features have obvious important implications for intratumoral therapy, the addition of immune adjuvants, such as KLH, have been used in other studies and may further enhance the anti-tumor effects of vaccination strategies [38-40]. Interestingly, in this report, our data showed that the addition of KLH in combination with the CCL21-DC may have a detrimental effect on elicitation of T cell responses given that it inhibited the ability of CCL21-DC to induce T cell proliferation and cytokine production. As a result, in the proposed clinical trial, KLH was removed from the protocol.

In support of our preclinical data in which the CCL21-DC have better anti-tumoral effects relative to AdCCL21 vector alone or recombinant CCL21 protein, the transduction of DC with AdCCL21 has the added advantage of immune enhancing effects presumably from the adenovirus sheltered within the DC. In our study, the supernatants from CCL21-DC significantly stimulated T2 cell chemotaxis. The rationale for utilizing T2 cells to evaluate the biological efficacy of CCL21-DC supernatants in the *in vitro *chemotaxis assays is an attempt to minimize the known experimental variability of human CD4^+ ^and CD8^+ ^T cells purified from different individuals, in contrast to the reproducible culturing and consistent T2 cell CCR7 expression of T2 cells. In vivo CD4^+ ^and CD8^+ ^T cells in fact may exhibit different expression of CCR7 based on their differentiation stage and antigen encounter [42-44]. Unexpectedly, the chemotactic effect of CCL21-DC supernatants was only partially inhibited by anti-CCL21 neutralizing antibody. The control vector transduced DC (CV-DC) in a similar fashion induced the chemotaxis in a CCL21 independent mechanism, suggesting that additional factors attributed to the adenovirus itself contributed to stimulation of chemotaxis. Thus, CCL21-DC induces chemotaxis via CCL21 dependent and independent mechanisms. While CCL21 is well known to induce chemotaxis of mature DC and naïve T cells, it has also been shown to be integral in the co-stimulation of naïve T cells and Th1 polarization of non-regulatory CD4^+^T cells [43]. Furthermore, CD4^+^CD25^+ ^regulatory T cells have been shown to be hypo-responsive to CCL21 induced migration [43]. A recent study by Tosello et al. [44] reported the differential expression of CCR7 in CD4^+ ^CD25^+ ^memory T regulatory cells identifying two populations: CCR7^+ ^central memory and CCR7^- ^effector memory T regulatory cells. While both subsets showed similar *ex vivo *effector function, only the CCR7^- ^effector memory population was predominant among T regulatory cells compared to conventional CD4^+ ^T cells [44]. Therefore, we anticipate that intratumoral delivery of CCL21-DC will induce effective chemotaxis *in vivo*, increase anti-tumor immune effector cells, and limit T regulatory cell trafficking.

Human lung cancer cells lack critical components of the antigen processing and presentation pathways required for initiation of a cell-mediated immune response [45]. Moreover, lung cancer patients have dramatically decreased numbers of circulating competent DC and their activity appears to be decreased by soluble factors in the tumor microenvironment [34,46]. A correlation between survival and the number of tumor-infiltrating DC has been reported [47], thus, activating DC at the tumor site may be an effective approach to treatment of NSCLC. Providing *ex vivo *generated DC may be a strategy to overcome some of these obstacles with the added advantage of DC cultured in GM-CSF and IL-4 may be more resistant to the suppressive effects of the tumor microenvironment [27,28].

## Conclusion

Viable and biologically active clinical grade CCL21 gene-modified DC can be effectively generated from cryopreserved leukapheresis products. We anticipate that clinical grade CCL21-DC may be an effective immunotherapeutic approach in the treatment of NSCLC, utilizing the *in vivo *tumor as the source of TAA, and thus eliminating the need for *ex vivo *priming with tumor antigens. On the basis of these pre-clinical findings, an intratumoral administration of clinical grade CCL21-transduced DC will be evaluated in a phase I clinical trial in late stage NSCLC patients.

## Competing interests

The authors declare that they have no competing interests.

## Authors' contributions

FB and HT equally contributed to the experimental design, data acquisition and analysis, and drafting of the manuscript. SH, SS, PSK, and RKB contributed to the critical revision of the manuscript. JL executed the titration of the clinical grade adenoviral vector. SMD, SS, and JML conceived the research project and critically revised the manuscript. SMD and JML gave the final approval of the version to be published. All authors have read and approved the final manuscript.
